# Fishbone foreign body ingestion in duodenal papilla: a cause of abdominal pain resembling gastric ulcer

**DOI:** 10.1186/s12876-020-01475-w

**Published:** 2020-10-02

**Authors:** Lizhi Yi, Zhengyu Cheng, Yafei Zhou, Qin Wang, Yangyang Liu, Ke Liu, Tao Wang, Xianfei Zhong

**Affiliations:** 1 Department of Gastroenterology, People’s Hospital of Leshan, Leshan, Sichuan People’s Republic of China; 2 Department of Anesthesiology, People’s Hospital of Leshan, Leshan, Sichuan People’s Republic of China; 3 Department of Radiology, People’s Hospital of Leshan, Leshan, Sichuan People’s Republic of China

**Keywords:** Fishbone, Foreign body, Duodenal papilla, Abdominal pain

## Abstract

**Background:**

Foreign body ingestion is a common clinical problem. The upper esophagus is the most common site of foreign body, accounting for more than 75% of all cases, but cases with a foreign body in the duodenal papilla or common bile duct are rarely reported.

**Case presentation:**

Herein, we report a rare case that a patient’s abdominal pain resembling gastric ulcer was caused by a 3 cm long fishbone inserted into the duodenal papilla.

**Conclusion:**

Fishbone inserted into the duodenal papilla can cause an abdominal pain resembling gastric ulcer. Endoscopy is useful for the diagnosis and treatment of fishbone ingestion in clinical.

## Background

Foreign body ingestion is a common diagnosis in clinical practice. Patients with foreign body ingestion usually present with odynophagia, dysphagia, the feeling of being stuck, chest or abdominal pain, vomiting and other symptoms [1]. The upper esophagus is the most common site of foreign body, accounting for more than 75% of all cases, but cases with a foreign body in the duodenal papilla or in the common bile duct are very rare [1, 2]. Here, for the first time we reported a 3 cm long fishbone inserted into the duodenal papilla, which resulted in the abdominal pain resembling gastric ulcer.

## Case presentation

**Fig. 1:**
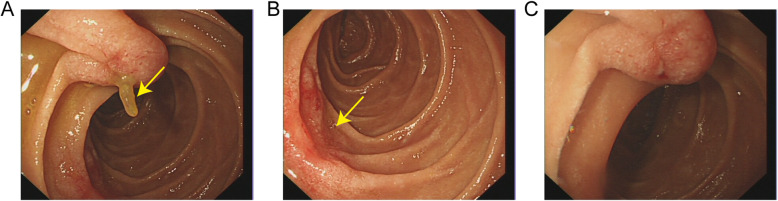
Endoscopic images before and after the removal of the foreign body: **a** Gastroscopy revealed something protruding from the duodenal papilla; **b** Patchy erosion below the duodenal papilla; **c** The duodenal papilla after extraction of the foreign body

**Fig. 2:**
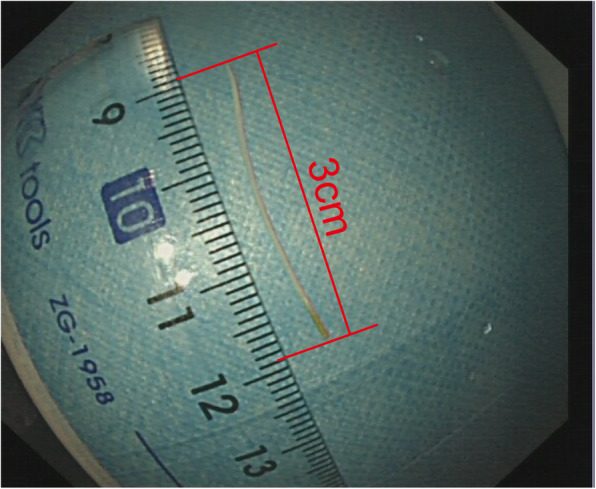
The fishbone successfully removed was about 3 cm long

A 52-year-old woman visited our hospital because of intermittent abdominal pain for half a month. She had no symptoms of melena, hematemesis or fever. The pain was localized to upper abdomen and regularly aggravated after taking meals. This patient had a gastric ulcer with bleeding cured 20 years ago. Besides that, she has no other significant medical history. Physical examination showed that there was a mild tenderness but not rebound tenderness in the upper abdomen. Owing to the history of gastric ulcer, a gastroscopy was performed, which indicated something protruding from the duodenal papilla with a patchy erosion below (Fig. 1a and b). Since the object was covered by mucus and bile, it was difficult to distinguish what it was. Based on this situation, we came up with the method that using a foreign body forceps to gently clamp the object. Thereafter, it was clamped and slowly pulled out from the duodenal papilla. To our surprise, it was a fishbone about 3 cm long (Fig. 2). After removing the foreign body, a small amount of blood was observed on the duodenal papilla (Fig. 1c), but there was no persistent bleeding after washing it with normal saline. After the operation, patient’s abdominal pain was relieved. She told us that she did eat fish half a month ago, but had no sense of being stuck. Subsequently, we suggested to carry out a CT scan of the abdomen because the fishbone had inserted into the duodenal papilla deeply for a long time, but the patient refused. One week later after the operation, the patient did not feel any discomfort and the abdominal pain was disappeared.

## Discussion and conclusion

Fishbone is the most common foreign body among the population in Asia, the Mediterranean, and other coastal countries due to dietary habits [3]. Generally, it is recommended to remove fishbone before they pass through the pylorus, otherwise 15–35% of them may perforate the intestine [4]. The frequent lodging sites of fishbones in the upper digestive tract are palatine tonsil, base of the tongue, valleculae, the piriform sinus and the first narrow area of esophagus [5]. It has been reported in a case that a toothpick was inserted into the common bile duct [6], but fishbone inserted into the duodenal papilla was never reported. Since the fishbone in our case was long and thin and very similar to a toothpick, we speculated that the fishbone might also be inserted into the common bile duct.

Patients with duodenal papillary foreign body may go through the acute stage and the chronic stage. At the acute stage, if there is a clear history of foreign body ingestion, the diagnosis is quick but the treatments may be different. Ouzhu M reported a dental prosthesis impacted in the duodenal papilla, which was then removed through endoscopy [7]. Dias R also reported a metal pin removed by a surgical exploration of the duodenum [8]. If the patients do not report a history of foreign body ingestion, both diagnosis and treatment will be difficult. Some foreign bodies may cause acute pancreatitis [9], while others may lead to ascending cholangitis [10]. In addition, at the acute stage, some foreign bodies might be misdiagnosed as choledocholithiasis [6]. At the chronic stage, some foreign bodies might form biliary stones, which may cause the cholangitis [11] or cholecystitis [12]. Moreover, the foreign body may also cause chronic pancreatitis [13].

Although the fishbone was successfully and safely removed using a foreign body forceps, we still believed that endoscopic ultrasonography might be a good choice before the operation. Because the patchy erosion under the foreign body suggested that the fishbone might penetrate deeply. Lack of experience, we didn’t realize it was needed until we saw the length of the fish bone. This was also what we learned from this case. In addition, liver enzymes tests were also recommended because the patient had a biliary foreign body. It was worth noting that gastroscopy should be the first examination for patients with foreign bodies. If the endoscopist failed to carefully observe the duodenal papilla, the patient’s diagnosis would be tortuous.

In our case, the patient’s pain was localized to upper abdomen and was regularly aggravated after taking meals, which was similarly to the pain caused by a gastric ulcer. We suspected that it may be related to the increased duodenal peristalsis after meals. And the patchy erosion below the duodenal papilla was very likely caused by the swaying fishbone. Therefore, the patients with regular abdominal pain, not only the stomach but also the duodenal papilla are needed to be carefully examined.

## Data Availability

Not applicable.

## Abbreviation

CT: Computed Tomography
